# Shoulder Isokinetic Strength Deficit in Patients with Neurogenic Thoracic Outlet Syndrome

**DOI:** 10.3390/diagnostics11091529

**Published:** 2021-08-24

**Authors:** Pauline Daley, Germain Pomares, Pierre Menu, Guillaume Gadbled, Marc Dauty, Alban Fouasson-Chailloux

**Affiliations:** 1 CHU Nantes, Service de Médecine Physique et Réadaptation Locomotrice et Respiratoire, 44093 Nantes, France; pauline.daley@chu-nantes.fr (P.D.); pierre.menu@chu-nantes.fr (P.M.); marc.dauty@chu-nantes.fr (M.D.); 2 CHU Nantes, Service de Médecine du Sport, 44093 Nantes, France; 3 Institut Européen de la Main, 2540 Luxembourg, Luxembourg; DrPomares@protonmail.com; 4 Medical Training Center, Hopital Kirchberg, 2540 Luxembourg, Luxembourg; 5 Inserm, UMR 1229, RMeS, Regenerative Medicine and Skeleton, Université de Nantes, ONIRIS, 44042 Nantes, France; 6 IRMS, Institut Régional de Médecine du Sport, 44093 Nantes, France; 7 CHU Nantes, Clinique Chirurgicale Orthopédique et Traumatologique, 44093 Nantes, France; guillaume.gadbled@chu-nantes.fr

**Keywords:** neurogenic, thoracic outlet syndrome, strength, endurance, isokinetic, QuickDASH

## Abstract

Neurogenic thoracic outlet syndrome (NTOS) is an impairing painful condition. Patients usually report upper-limb pain, weakness and paresthesia. Shoulder weakness is frequently reported but has never been described with objective strength evaluation. We aimed to compare isokinetic shoulder strength between patients with NTOS and healthy controls. Patients and controls were prospectively evaluated with an isokinetic strength test at 60 and 180°/s, and an endurance test (30 repetitions at 180°/s) of the shoulder rotators. Patients were functionally assessed with QuickDASH questionnaires. One hundred patients and one hundred healthy subjects were included. Seventy-one percent of patients with NTOS were females with a mean age of 39.4 ± 9.6. They were compared to controls, 73% females and the mean age of 38.8 ± 9.8. Patients’ mean QuickDASH was 58.3 ± 13.9. Concerning the peak of strength at 60°/s, the symptomatic limbs of patients with NTOS had significantly 21% and 29% less strength than the control limbs for medial and lateral rotators, respectively (*p* ≤ 0.001). At 180°/s, the symptomatic limbs had significantly 23% and 20% less strength than the controls for medial and lateral rotators, respectively (*p* ≤ 0.001). The symptomatic limbs had significantly 45% and 30% less endurance than the controls for medial and lateral rotators, respectively (*p* ≤ 0.001). These deficits were correlated to the QuickDASH. Patients with NTOS presented a significant deficit of strength and endurance of the shoulder rotators correlated to disability. This highlights the interest in upper-limb strength evaluation in the diagnostic process and the follow-up of NTOS.

## 1. Introduction

The thoracic outlet syndrome (TOS) is a chronic painful condition related to the compression of the upper-limb neurovascular bundle [1]. Three distinct manifestations are usually considered: neurogenic TOS (NTOS), venous TOS and arterial TOS [2,3]. NTOS represents the most common form in more than 90% of the cases [1,2,4], and it is secondary to intermittent compression of the brachial plexus at the supraclavicular scalene triangle and between the first rib and the clavicle, or at the sub-coracoid space levels [4,5,6]. NTOS is responsible for functional disability and has a social impact [5,7]. Women are affected in about 70% of the cases, around 20 to 40 years old [5,8]. Patients describe upper-limb pain, paresthesia and weakness, especially during prolonged elevated arm position or during repetitive upper-limb movements. Its diagnosis is still difficult due to the lack of specificity of the symptoms and the clinical/radiological exams [3,5,9,10,11]. However, a more consensual diagnosis of NTOS is now possible thanks to specific guidelines [3,5,12,13].

Upper-limb weakness is one of the symptoms frequently reported by patients. Recently, Fouasson-Chailloux et al. [14] have confirmed objectively a significant hand strength deficit in patients with NTOS both on the symptomatic and the asymptomatic hands, highlighting the interest of strength evaluation in the diagnosis process and the follow-up of NTOS [3,12]. However, patients often complain of shoulder weakness and fatigability, which is rarely assessed in clinical practice. Indeed, only one study has evaluated shoulder flexion strength and fatigue in a small and heterogeneous group of patients with TOS compared to controls [15]. Results were difficult to interpret due to methodological issues. However, isokinetic assessment is an objective evaluation that has shown its interest to evaluate muscular strength and endurance [16]. It is accurate and reliable to assess shoulder strength and endurance, especially for medial and lateral rotator muscles [16,17,18].

In this study, we aimed to compare the isokinetic strength of the shoulder rotators between NTOS patients and healthy controls. We hypothesized that patients with NTOS would have an objective shoulder strength deficit compared to controls.

## 2. Materials and Methods

### 2.1. Patients

All the participants were recruited from 21 July 2020 to 11 July 2021. We included patients addressed by upper-limb surgeons (vascular surgeons, orthopedists or hand surgeons), rheumatologists or vascular physicians for a specific protocol of NTOS rehabilitation. This rehabilitation program was proposed to the most disabled patients in the case of ineffective outpatient physiotherapy, especially in the case of prolonged work stopping (>3 months). Patients should report a severe quality of life impairment. The rehabilitation program consisted of inpatient hospitalization for 3 to 4 weeks. Before the beginning of the program, all the patients performed routinely a strength assessment, including shoulder isokinetic testing of the rotator cuff. To be included, patients should fulfill the diagnostic criteria for unilateral or bilateral NTOS according to the Consortium for Research and Education on thoracic outlet syndrome (Table 1) [5,12]. They also should be over 18 years old. Exclusion criteria were: (1) previous neck or upper-limb surgery, (2) upper-limb musculoskeletal disorders (rotator-cuff tendinopathy, osteoarthritis for example), (3) cervicobrachial neuralgia, (4) other entrapment neuropathy of the upper limb (all the patients with NTOS underwent electrodiagnostic testing [19]), (5) any contraindication to the isokinetic testing.

The protocol study was approved by the Committee of Ethics “Comité de Protection des Personnes d’Ile-de-France II”, and all the patients and healthy controls gave their verbal consent to participate in the study. No written consent was needed for the participants because the study did not modify patients’ usual care; the procedure had minor risks for healthy subjects. The study was registered on ClinicalTrials.gov (accessed on 25 July 2021): NCT04145778.

### 2.2. Healthy Control Subjects

Healthy controls were volunteers recruited among the University Hospital staff. Criteria of exclusion were: history of neck or shoulder surgery, history of rotator cuff tendinopathy, upper-limb neurologic disorder, and high-level or elite athletes.

### 2.3. Isokinetic Testing

After a 5 min warm up with an arm cranking ergometer (Ergoselect^®^ 400, Ergoline, Bitz, Germany), isokinetic strength tests were performed using a Humac Norm^®^ dynamometer (CSMI, Medimex, Sainte-Foy-lès-Lyon, France). The medial and lateral rotations were performed in the scapular plane [16,20,21,22]: the subjects were seated, hips bent at 85° to the trunk in order to limit spine compensations. The seat rotation was 35° relative to the dynamometer in order to place the shoulder in the plane of the scapula. The dynamometer was tilted at 40° from the horizontal plane. The seat position was adjusted so that the subjects’ arm was placed in the plane of the scapula, the 2 shoulders were aligned in a horizontal line, and the axis of the arm was in line with the dynamometer. The elbow was bent at 90°, and the prono-supination was neutral. The amplitudes of the medial and lateral rotations of the shoulder were fixed at 65 and 35°, respectively. The 2 shoulders were evaluated in a random order in the case of bilateral NTOS and for the healthy subjects. In the case of unilateral NTOS, the non-symptomatic shoulder was tested first. After familiarization with the isokinetic movement (5 sub-maximal repetitions at 240°/s), the subjects were tested with 3 repetitions of concentric medial and lateral shoulder rotations at 60°/s, followed by an isokinetic endurance test of 30 concentric repetitions at 180°/s. Recovery between the series was 20 s.

### 2.4. Isokinetic Test Interpretation

Several isokinetic parameters were taken into consideration: relative peak torque defined by the maximal peak torque normalized to the body mass (Nm/kg) at the 2 angular speeds (60°/s and 180°/s); Isokinetic endurance parameters: relative shoulder medial and external rotators total work normalized to body mass (J/kg). The reliability previously established by intra-class correlation coefficient (ICC) of the concentric isokinetic peak torque at 60°/s is excellent on medial rotation (ICC: 0.93 on left side; 0.94 on right side) and lateral rotation (ICC: 0.92 on left side; 0.95 on right side) [17]. We also established in our studied population of volunteers the reproducibility of the concentric isokinetic peak torque at 180°/s (ICC medial rotation: 0.98 on left side; 0.97 on right side; ICC lateral rotation: 0.91 on left side; 0.92 on right side) and the total work at 180°/s (ICC medial rotation: 0.86 on left side; 0.85 on right side; ICC lateral rotation: 0.83 on left side; 0.85 on right side). These results are good to excellent [23].

### 2.5. QuickDASH Questionnaire

At the beginning of the rehabilitation program, patients with NTOS completed a French version of the QuickDASH [24], a short version of the DASH questionnaire (Disabilities of the shoulder and Arm) [25]. It comprises 11 questions evaluating upper-limb function and symptoms. The score ranges from 0 (no disability) to 100 (most severe disability). The QuickDASH has frequently been used in the evaluation of patients with NTOS [14,26,27,28,29]. In the case of bilateral NTOS, patients were asked to assess their most disabling upper-limb.

### 2.6. Pain Assessment

The pain was evaluated for all the patients with a numerical rating scale (NRS) ranging from 0 (no pain) to 10 (worst pain possible) [14,30]. In the case of bilateral NTOS, the most painful upper-limb was assessed.

### 2.7. Statistical Analysis

Statistical analyses were performed with IBM SPSS 23.0 software (Armonk, NY, USA). Quantitative parameters were presented as mean and standard deviation. The Kolmogorov–Smirnov test was used to assess the normality of the data. Quantitative variable comparisons between patients with NTOS and healthy controls were performed with independent t-tests for independent variables or with Mann–Whitney tests (if data were not normally distributed), and qualitative comparisons were performed with χ^2^ tests. Taking the upper-limbs as units, we performed a Kruskal–Wallis test followed by a Dunn post hoc test to compare asymptomatic upper-limbs to symptomatic upper-limbs in patients with NTOS and to both upper-limbs of healthy controls, considering each upper-limb independently. Spearman’s correlation coefficients (*R*^2^) were calculated to assess the association between the isokinetic shoulder strength in patients with unilateral NTOS and the QuickDASH score, the duration of the symptoms and the pain. This calculation was not possible in bilateral NTOS because only one QuickDASH per patient was achieved, the duration of symptoms was considered for the oldest diagnosis, and only one NRS was assessed. The correlation coefficient interpretation was [31]: strong correlation (*R*^2^ > 0.9); high (0.7 < *R*^2^ < 0.9); moderate (0.5 < *R*^2^ < 0.7), low (0.3 < *R*^2^ < 0.5), negligible (*R*^2^ < 0.3). The level of significance was considered at *p* < 0.05.

## 3. Results

### 3.1. Participants’ Characteristics

One hundred patients addressed for a specialized NTOS rehabilitation were included. Seventy-one percent were females, and their mean age was 39.4 ± 9.6 years old. They had a mean QuickDASH score of 58.3 ± 13.9 (ranging from 13.6 to 88.6). The duration of their symptoms was 38.0 ± 34.2 months. Sixty-seven patients suffered from unilateral NTOS, and thirty-three complained of bilateral symptoms. Their pain on NRS was 5.4 ± 1.7. None of the patients presented an objective strength deficit on the manual muscle testing (Medical Research Council), nor muscular atrophy. The control group was composed of 100 healthy volunteers with 73% of females and a mean age of 38.8 ± 9.8. Both groups were comparable in terms of sex (*p* = 0.75), age (*p* = 0.40), weight (*p* = 0.12) and height (*p* = 0.11). However, body mass index (BMI) was significantly higher for NTOS patients compared to controls, 25.2 ± 4.7 vs. 23.5 ± 3.5 kg/m^2^, respectively (*p* = 0.02). Comparisons between both groups are presented in Table 2.

Nineteen patients had abnormalities on the electrodiagnostic testing, compatible with NTOS diagnosis: six brachial plexopathies and thirteen abnormalities of the medial antebrachial cutaneous nerve conduction (out of 23 evaluations of this nerve).

No difference was found between patients with unilateral and bilateral NTOS, for sex (*p* = 0.46) for age, 38.7 ± 10.2 vs. 41.0 ± 8.3, respectively (*p =* 0.27), for height, 167.5 ± 9.1 vs. 166.5 ± 8.9, respectively (*p* = 0.61), for weight, 70.5 ± 15.0 vs. 70.9 ± 16.1, respectively (*p* = 0.90), and for BMI, 25.1 ± 4.6 vs. 25.5 ± 4.9, respectively (*p =* 0.68). The mean QuickDASH score was 57.5 ± 14.9 for patients with unilateral NTOS and 59.9 ± 11.9 for those with bilateral symptoms (*p* = 0.63). The symptoms’ duration was also not significantly different, with a mean duration of 33.2 ± 27.0 months for patients having unilateral symptoms vs. 47.6 ± 44.4 for patients having bilateral symptoms (*p* = 0.36). The NRS for pain was not different between the unilateral NTOS patients and those with bilateral symptoms, 5.6 ± 1.6 vs. 5.8 ± 1.8 (*p* = 0.62).

### 3.2. Isokinetic Testing

The distribution of dominant and non-dominant upper-limbs was not statistically different between symptomatic, asymptomatic, and control limbs (*p* = 0.08) (Table 3).

Concerning the peak torque at 60°/s, the symptomatic limbs of patients with NTOS had significantly 21% and 29% less strength than the control limbs for medial and lateral rotators, respectively (0.46 ± 0.16 vs. 0.58 ± 0.15 Nm/kg, *p* ≤ 0.001, and 0.20 ± 0.09 vs. 0.28 ± 0.09 Nm/kg, *p* ≤ 0.001, respectively). The asymptomatic limbs also had significantly 10% and 18% less strength than the control limbs for medial and lateral rotators, respectively (0.52 ± 0.16 vs. 0.58 ± 0.15 Nm/kg, *p* ≤ 0.01, and 0.23 ± 0.09 vs. 0.28 ± 0.09 Nm/kg respectively, *p* ≤ 0.01).

Concerning the peak torque at 180°/s, the symptomatic limbs of patients with NTOS had significantly 23% and 20% less strength than the control limbs for medial and lateral rotators respectively (0.41 ± 0.16 vs. 0.53 ± 0.14 Nm/kg, *p* ≤ 0.001, and 0.20 ± 0.07 vs. 0.25 ± 0.07 Nm/kg, *p* ≤ 0.001). The asymptomatic limbs also had significantly 15% and 12% less strength than the control limbs for medial and lateral rotators, respectively (0.45 ± 0.15 vs. 0.53 ± 0.14 Nm/kg, *p* ≤ 0.001, and 0.22 ± 0.09 vs. 0.25 ± 0.07 Nm/kg respectively, *p* ≤ 0.01).

Concerning endurance test (total work for 30 repetitions at 180°/s), the symptomatic limbs of patients with NTOS had significantly 45% and 54% less endurance than the control limbs for medial and lateral rotators respectively (10.0 ± 6.1 vs. 18.1 ± 5.5 J/kg, *p* ≤ 0.001, and 2.49 ± 2.2 vs. 5.4 ± 2.9 J/kg, *p* ≤ 0.001, respectively). The asymptomatic limbs presented also significantly 30% and 34% less endurance than the control limbs for medial and lateral rotators respectively (12.75 ± 6.0 vs. 18.1 ± 5.5 J/kg, *p* ≤ 0.001, and 3.56 ± 2.7 vs. 5.4 ± 2.9 J/kg respectively, *p* ≤ 0.001).

No difference was found between the symptomatic limbs and the asymptomatic limbs for the peak torque and the endurance of medial rotators. However, the symptomatic limbs presented 30% less endurance than the asymptomatic limbs on lateral rotators (2.49 ± 2.2 vs. 3.56 ± 52.7 J/kg, *p* ≤ 0.01). All the isokinetic results are presented in Table 3.

### 3.3. Correlation between Limb Strength and Symptoms in Patients with Unilateral NTOS

QuickDASH had an inverse low correlation with all isokinetic results for strength and endurance. Indeed, there was a significantly low inverse correlation between medial rotators at 60°/s, lateral rotators at 60°/s and QuickDASH (−0.434, *p* ≤ 0.001, and −0.465, *p* ≤ 0.001, respectively). There was also a significantly low inverse correlation between medial rotators at 180°/s, lateral rotators at 180°/s and QuickDASH (−0.362, *p* ≤ 0.01 and −0.401, *p* ≤ 0.001, respectively). QuickDASH had a low inverse correlation with endurance in medial rotators and lateral rotators (−0.347, *p* ≤ 0.01 and −0.341, *p* ≤ 0.01, respectively).

The pain was also slightly and inversely correlated to strength and endurance. Indeed, there was a significantly low inverse correlation between medial rotators at 60°/s, lateral rotators at 60°/s and NRS (−0.358, *p* ≤ 0.01, and −0.399, *p* ≤ 0.001, respectively). There was also a significantly low inverse correlation between medial rotators at 180°/s, lateral rotators at 180°/s and NRS (−0.317, *p* ≤ 0.01 and −0.399, *p* ≤ 0.001, respectively). The pain was lowly and inversely correlated to the endurance of the medial and lateral rotators (−0.355, *p* ≤ 0.01 and −0.366, *p* ≤ 0.01, respectively). There was a significant moderate correlation between QuickDASH and pain (0.558, *p* ≤ 0.001).

No correlation was found between the symptoms’ duration and isokinetic findings and between the symptoms’ duration and QuickDASH. Table 4 reports the correlation between isokinetic findings and clinical features.

## 4. Discussion

In this original study, we studied proximal muscles of the upper-limb in patients with NTOS. The shoulder evaluation appears consistent with patients’ symptomatology, despite not being systematically assessed in clinical practice, both during the diagnostic process and the follow-up of patients with NTOS. Indeed, only distal strength deficit is considered for the diagnosis of NTOS [12] and has been objectively described in this population [14]. In fact, in a recent study, Fouasson-Chailloux et al. [14] showed a significant hand strength deficit in patients with NTOS, both on the key pinch and the hand grip compared to controls or to contralateral hands for unilateral NTOS. However, this study did not assess the proximal weakness of the upper-limbs, which is commonly described by patients.

In this work, we chose to compare symptomatic upper-limbs with asymptomatic sides for patients presenting unilateral NTOS and given previous data indicating a strength deficit in key pinch and grip for asymptomatic limbs of unilateral NTOS [14]. We found many shoulder strength and endurance deficits. These strength and endurance deficits may be due to the NTOS itself. Indeed, Braun et al. [32] have previously shown that scalene muscle blocks improve work product performance of the upper-limb from 93% to 106% and increase the delay of fatigue from 50% to 66%. However, other reasons may be discussed to explain our findings. Firstly, we could not exclude that pain might have influenced our results, especially because we found a significant inverse correlation between the strength, the endurance and the NRS (from −0.317 to −0.399, *p* ≤ 0.01). However, this correlation was low, which means that despite a statistically significant implication, pain has a weak influence on strength and endurance. Moreover, asymptomatic shoulders also exhibited significantly less strength and endurance than control shoulders. Secondly, this bilateral deficit may have been influenced by the chronic pain in NTOS, as previously mentioned [14]. Indeed, some unilateral chronic upper-limb painful conditions such as epicondylalgia are responsible for a contralateral decrease of the sensorimotor performances [33,34,35,36]. For example, Alizadehkhaiyat et al. reported a 16% to 29% strength deficit on the different upper-limb muscles of the contralateral side compared to those of the controls [36]. This phenomenon may be explained by a reverse effect of “cross education”. In fact, it has been previously studied that unilateral exercises were responsible for a significant increase of strength and skills on the contralateral side. Therefore, we may assume that the reduction of the use of one side can impact negatively the other side [34]. These results may also be explained by a persistent reduction of the pain-related interhemispheric inhibition occurring from the affected to the unaffected motor cortex [37]. However, this hypothesis needs further studies to confirm the role of chronic pain in this contralateral upper-limb strength deficit; the use of transcranial magnetic stimulation could be of interest in this way of research [34]. Thirdly, the prolonged duration of the pathology could also be put forward to explain these deficits because of an under solicitation of the upper-limbs, as previously shown in rheumatoid arthritis with a correlation between the strength deficit and the disease duration [38]. However, we may reject this point in our study because we found no correlation between the deficit of strength or endurance and the delay of the evolution of the symptoms. Finally, we cannot exclude that some patients with unilateral NTOS had also contralateral symptoms not declared because of a lesser impairment. However, patients were included after a rigorous clinical examination to prevent such cases.

To the best of our knowledge, only one study performed isokinetic evaluations of patients with TOS [15]. In that study, Ozçacar et al. showed a tendency to a higher fatigability on the shoulder flexors in patients with TOS. However, their study included only 23 patients compared to 15 healthy individuals, and the evaluation was made on shoulder flexors, which is unusual in isokinetic evaluation and may represent an important issue concerning the reproducibility of the results [15]. Moreover, they included patients with TOS but without clearly defining the type TOS, which may have been responsible for a heterogeneity in their group. On the contrary, in our study, all the patients included had a diagnosis of NTOS based on CORE-TOS criteria [12]. Furthermore, our patients were similar in terms of mean age and sex ratio to previous studies including patients with NTOS [39,40], although in some other studies the age can be slightly lower by a few years [5,8,29]. This small difference may be due to divergences in the management strategy of NTOS within the centers, in particular the delay of access to the surgery. The sitting position used for isokinetic evaluation is relevant as it does not trigger pain, which was an interesting criterion for our painful population, and it also minimizes compensation phenomenon, and it is commonly adopted to evaluate shoulder rotators [16,20]. The dominance effect is usually negligible in shoulders [17,41], especially in our population because there was no significant difference of repartition between dominant and non-dominant upper-limbs between groups (*p* = 0.08), which prevent any misinterpretations of the strength evaluation. Several methodologies have been described to assess isokinetically muscle endurance and fatigability. Indeed, some studies have described isokinetic evaluation of the fatigue by calculating a fatigue ratio, as the ratio between the last and the first third of work [42,43]. We evaluated a slightly different measure, with total work on 30 repetitions. Indeed, total work reflects endurance on a large number of repetitions [44,45,46]. Kannus et al. [47] concluded that these two methods had similar meanings concerning endurance evaluation at the lower limbs. However, we assume that overall endurance with total work reduce the importance of the regain of strength in the last repetitions, which is a common observation in clinical practice, especially in patients not familiar with isokinetic testing.

In this study, we used the QuickDASH questionnaire to assess the upper-limb disability of the patients with NTOS because it is validated in the French language and has an excellent test-retest reliability [24]. Our mean QuickDASH scores for uni- and bilateral NTOS were consistent with previous studies using this questionnaire, from 52.9 to 62.6 [27,28,29]. We showed a significant negative correlation between shoulder strength and QuickDASH in our patients, which is consistent with previous results finding a correlation with hand strength [14]. These results confirm that disability is correlated to the deficit of strength, although this is only a weak correlation. Rehabilitation programs may target strength evolution more specifically during the follow-up of patients with NTOS to assess if muscular strengthening could improve upper-limb function. Moreover, the upper-limb strength and endurance assessment could be an interesting parameter to monitor the impact of the rehabilitation program or the surgical management on weakness [48].

This study has limitations. Indeed, our study concerned only the most disabled patients with NTOS addressed to rehabilitation because of ineffective outpatient physiotherapy, which prevents generalization of the results. Furthermore, the patients might have a lower level of activity compared to controls, which could have explained the reduction of strength and endurance, especially on the asymptomatic upper-limbs. To limit this bias, we have excluded controls with a high level of activity. Moreover, correlations between isokinetic shoulder results and, QuickDASH, pain and symptoms duration were only performed in the group of patients with unilateral NTOS because these clinical evaluations were only assessed once. However, these parameters were comparable between patients with unilateral and bilateral NTOS, which may make the results generalizable to both groups. Another limit is the significant difference of BMI in our patients compared to controls despite comparability of the heights and weights, which might have minimized the comparability between our two groups. However, all our values were normalized to body mass (Nm/kg) to prevent difficulties of comparison.

## 5. Conclusions

Patients with NTOS present a muscular deficit of the shoulder rotators both on strength and endurance compared to healthy controls. These findings appear consistent with clinical observations. The strength deficit is lowly but significantly correlated to their disability and the pain intensity. These results allow a better understanding of NTOS and offer perspectives for exhaustive evaluation and management of NTOS. Lack of strength can be underestimated in unilateral NTOS when compared to the contralateral side. The contralateral side should be thoroughly examined in order to look for bilateral weakness. The treatment, especially rehabilitation, should endeavour to consider the asymptomatic upper-limb as well as the symptomatic one in the case of unilateral NTOS. Future studies are necessary to evaluate the evolution of these shoulder strength and endurance deficits after conservative or surgical treatment.

## Figures and Tables

**Table 1:** Diagnosis criteria for Neurogenic thoracic outlet syndrome (NTOS) according to the Consortium for Research and Education on thoracic outlet syndrome [5,12].

Diagnosis Criteria for NTOS
No other probable diagnosis Symptoms duration ≥ 12 weeks
Principal symptoms 1 a: Pain in the neck, upper back, shoulder, arm, and/or hand. 1 b: Numbness, paresthesia, and/or weakness in the arm, hand, or digits.
Symptom characteristics 2 a: Pain/paresthesia/weakness exacerbated by elevated arm positions. 2 b: Pain/paresthesia/weakness exacerbated by prolonged or repetitive arm/hand use. 2 c: Pain/paresthesia radiate down the arm from the supraclavicular or infra clavicular spaces.
Clinical History 3 a: Symptoms began after occupational, recreational, or accidental injury of the head, neck, or upper extremity, including repetitive upper extremity strain or overuse. 3 b: Previous ipsilateral clavicle or first rib fracture or known cervical rib. 3 c: Previous cervical spine or ipsilateral peripheral nerve surgery without sustained improvement in symptoms. 3 d: Previous conservative or surgical treatment for ipsilateral TOS.
Physical examination 4 a: Local tenderness on palpation over the scalene triangle and/or sub-coracoid space. 4 b: Arm/hand/digit paresthesia on palpation over the scalene triangle and/or sub-coracoid space. 4 c: Objectively weak handgrip, intrinsic muscles, or digit 5, or thenar/hypothenar atrophy.
Provocative maneuvers 5 a: Positive upper limb tension test (ULTT). 5 b: Positive 3-min elevated arm stress test (EAST).
NTOS diagnosis was retained if patients had upper limb symptoms present for at least 12 weeks, which extend beyond the distribution of a single cervical nerve root or peripheral nerve, had not been satisfactorily explained by another condition, and met at least one criterion in at least four of five categories: (1) Principal symptoms, (2) Symptom characteristics, (3) Clinical History, (4) Physical examination, and (5) Provocative maneuvers.

**Table 2:** Comparison between patients with neurogenic TOS and healthy controls.

	Patients with NTOS *n* = 100	Healthy Controls *n* = 100	*p*
Sex:female, *n* (%) male, *n* (%)	71 (71%) 29 (29%)	73 (73%) 27 (27%)	0.75 ^a^
Age, years ± SD (min-max)	39.4 ± 9.6 (22–61)	38.8 ± 9.8 (24–67)	0.40 ^b^
Height, cm ± SD (min-max)	167.2 ± 9.0 (150–192)	169.2 ± 8.4 (152–190)	0.11 ^c^
Weight, kg ± SD (min-max)	70.6 ± 15.3 (43–106)	67.6 ± 12.9 (48–112)	0.12 ^c^
Body Mass Index, kg/m^2^ ± SD (min-max)	25.2 ± 4.7 (17.0–39.9)	23.5 ± 3.5 (17.0–35.4)	0.02 ^b^

NTOS: neurogenic thoracic outlet syndrome; SD: standard deviation. ^a^: χ^2^ test; ^b^: Mann–Whitney test, ^c^: Independent *t*-test.

**Table 3:** A comparison of isokinetic shoulder strength between symptomatic limbs, asymptomatic limbs in patients with NTOS and limb controls (Kruskal–Wallis test followed by Dunn post hoc test).

	Symptomatic Limbs *n* = 133	Asymptomatic Limbs *n* = 67	Control Limbs *n* = 200	H	*p*
Dom/non-Dom, *n*	74/59	26/41	100/100		0.08 ^#^
MR 60°/s, Nm/kg	0.46 ± 0.16 ^a^***	0.52 ± 0.16 ^b^**	0.58 ± 0.15 ^a^***^, b^**	46.6	<0.0001
LR 60°/s, Nm/kg	0.20 ± 0.09 ^a^***	0.23 ± 0.09 ^b^**	0.28 ± 0.09 ^a^***^, b^**	56.9	<0.0001
MR 180°/s, Nm/kg	0.41 ± 0.16 ^a^***	0.45 ± 0.15 ^b^***	0.53 ± 0.14 ^a^***^, b^***	48.5	<0.0001
LR 180°/s, Nm/kg	0.20 ± 0.07 ^a^***	0.22 ± 0.09 ^b^**	0.25 ± 0.07 ^a^***^, b^**	45.3	<0.0001
MR endurance, J/kg	10.0 ± 6.1 ^a^***	12.75 ± 6.0 ^b^***	18.1 ± 5.5 ^a^***^, b^***	125.9	<0.0001
LR endurance, J/kg	2.49 ± 2.2 ^a^***^, c^**	3.56 ± 2.7 ^b^***^, c^**	5.4 ± 2.9 ^a^***^, b^***	105.2	<0.0001

Dom: dominant upper-limb; non-Dom: non-dominant upper-limb; MR 60°/s: medial rotators at 60°/s; LR 60°/s: lateral rotators at 60°/s; MR 180°/s: medial rotators at 180°/s; LR 180°/s: lateral rotators at 180°/s; Nm: Newton-meter; SD: standard deviation; ^#^: χ^2^ test for the 3 populations. ^a^: Significant difference between symptomatic limbs and controls; ^b^: Significant difference between asymptomatic limbs and controls; ^c^: Significant difference between symptomatic limbs and asymptomatic limbs. Dunn’s test: ** *p* ≤ 0.01; *** *p* ≤ 0.001. H: H-statistic of the Kruskal-Wallis test.

**Table 4:** Spearman’s correlation between isokinetic shoulder strength and endurance and clinical features of patients with unilateral NTOS (*n* = 67).

	QuickDASH	Pain (NRS)	Symptoms Duration
MR 60°/s	−0.434 ***	−0.358 **	0.189
LR 60°/s	−0.465 ***	−0.399 ***	0.113
MR 180°/s	−0.362 **	−0.317 **	0.115
LR 180°/s	−0.401 ***	−0.399 ***	0.121
MR endurance	−0.347 **	−0.355 **	0.126
LR endurance	−0.341 **	−0.366 **	0.033
QuickDASH	1	0.558 ***	−0.052

MR 60°/s: medial rotators at 60°/s; LR 60°/s: lateral rotators at 60°/s; MR 180°/s: medial rotators at 180°/s; LR 180°/s: lateral rotators at 180°/s. Spearman’s correlation: ** *p* ≤ 0.01; *** *p* ≤ 0.001.

## Data Availability

The data presented in this study are available on request from the corresponding author. The data are not publicly available due to ethical reasons.
